# Direct neural evidence for the contrastive roles of the complementary learning systems in adult acquisition of native vocabulary

**DOI:** 10.1093/cercor/bhab422

**Published:** 2021-12-07

**Authors:** Katherine R Gore, Anna M Woollams, Stefanie Bruehl, Ajay D Halai, Matthew A Lambon Ralph

**Affiliations:** Division of Neuroscience and Experimental Psychology, School of Biological Sciences, University of Manchester, Manchester M13 9GB, UK; Division of Neuroscience and Experimental Psychology, School of Biological Sciences, University of Manchester, Manchester M13 9GB, UK; Division of Neuroscience and Experimental Psychology, School of Biological Sciences, University of Manchester, Manchester M13 9GB, UK; St Mauritius Rehabilitation Centre, Meerbusch & Heinrich-Heine University, 40225 Duesseldorf, Germany; Clinical and Cognitive Neurosciences, Department of Neurology, Medical Faculty, RWTH Aachen University, 52074 Aachen, Germany; MRC Cognition & Brain Sciences Unit, University of Cambridge, Cambridge CB2 7EF, UK; MRC Cognition & Brain Sciences Unit, University of Cambridge, Cambridge CB2 7EF, UK

**Keywords:** aging, fMRI, language, semantics, vocabulary learning

## Abstract

The Complementary Learning Systems (CLS) theory provides a powerful framework for considering the acquisition, consolidation, and generalization of new knowledge. We tested this proposed neural division of labor in adults through an investigation of the consolidation and long-term retention of newly learned native vocabulary with post-learning functional neuroimaging. Newly learned items were compared with two conditions: 1) previously known items to highlight the similarities and differences with established vocabulary and 2) unknown/untrained items to provide a control for non-specific perceptual and motor speech output. Consistent with the CLS, retrieval of newly learned items was supported by a combination of regions associated with episodic memory (including left hippocampus) and the language-semantic areas that support established vocabulary (left inferior frontal gyrus and left anterior temporal lobe). Furthermore, there was a shifting division of labor across these two networks in line with the items’ consolidation status; faster naming was associated with more activation of language-semantic areas and lesser activation of episodic memory regions. Hippocampal activity during naming predicted more than half the variation in naming retention 6 months later.

## Introduction

Across the lifespan, humans need to acquire new knowledge and do so rapidly with relative ease. One lifelong learning process is vocabulary acquisition. Beyond the initial influx of new language in childhood, there are numerous words, meanings, and expressions to learn throughout adulthood. Thus, individuals constantly acquire new vocabulary relating to their everyday lives, hobbies, and profession. Re-establishing vocabulary is also a key target for those with language impairment (aphasia) after brain damage from injury, stroke, or dementia because word-finding difficulties (anomia) are a pervasive and frustrating feature of all types of aphasia (Rohrer et al. 2008). Therefore, from both cognitive and clinical neuroscience perspectives, it is fundamentally important to understand both the cognitive and neural bases of vocabulary acquisition.

One influential theory is the Complementary Learning Systems (CLS; Marr 1971; McClelland et al. 1995) model. This theory proposes that new knowledge is initially coded through rapidly formed, sparse representations supported by the medial temporal lobes (MTL) and hippocampus. Longer-term consolidation and evolution of generalizable representations follow from slower, interleaved learning and MTL replay to neocortical regions. Thus, over time, there is a gradual shift in the division of representational load between MTL and neocortical regions (with the rate of change depending on various factors: cf. McClelland et al. 2020). The CLS provides a potentially generalizable theoretical framework for the acquisition of many different kinds of knowledge including language acquisition (cf. Davis and Gaskell 2009). There is, however, little direct neural evidence for this theory in long-term language learning, particularly in adults who already have large and varied vocabularies.

To date, few if any studies have explored the processes that underpin new vocabulary learning within adults’ native language (i.e., learning the meaning and name of novel items/concepts as one might do when learning about a new hobby, profession, or technology). Instead, the handful of pre-existing investigations has typically focused on second language learning. Studies have adopted different experimental designs. Some have required participants to link brand new names to pre-existing, well-established meanings (Raboyeau et al. 2004; Yang et al. 2015). Alternatively, to avoid the unfamiliar phonetic and phonological elements of second languages, researchers have used pseudowords that conform to the phonological structure of the native language (Mestres-Missé et al. 2008; Davis et al. 2009; Paulesu et al. 2009; Ozubko and Joordens 2011; Pohl et al. 2017). Pseudowords, however, do not have semantic meanings to aid learning and consolidation. Takashima et al. (2017) trained participants with pseudowords, half with word meanings, to explore this issue. Participants completed a same-day and 1-week later recognition functional magnetic resonance imaging (fMRI) task. Novel words with semantic information at encoding were better retained but utilized both the episodic and semantic systems during recognition at both stages. Of course, learning additional names for pre-existing items may generate competition between new and old words when naming. This proactive interference can skew accuracy and reaction times (RTs; Gaskell and Dumay 2003). To avoid these issues, researchers sometimes use abstract (i.e., meaningless) images alongside pseudowords (Takashima et al. 2014).

Although these pseudoword studies assessed performance through recognition tasks rather than the full recall process needed in speech production, they nevertheless indicate some important target brain regions for investigating native vocabulary. In an online fMRI associative learning study, Breitenstein et al. (2005) presented participants with an image and paired auditory pseudoword. Participants learned the novel vocabulary through associative learning exposure, with higher occurrences of “correct” pairings. There was strong evidence of initial hippocampal encoding of pseudowords. In addition, there was hippocampal modulation during online pseudoword learning, whereby a linear decrease of left hippocampal activity paralleled increases in pseudoword accuracy over the training. Davis et al. (2009) used fMRI to measure neural responses to novel pseudowords at different stages of consolidation. Unfamiliar novel words had elevated hippocampal responses, and this response correlated with post-scanning measures of word learning. Similar to Breitenstein et al. (2005), as participants completed more training, there were associated hippocampal activity decreases. These studies provide evidence for the first stage of the CLS, in short-term learning of pseudowords. Such studies also provide second-stage neocortical regions of interest (ROIs), with differential responses to novel and existing words, including the left temporal lobe (Raboyeau et al. 2004; Davis et al. 2009), bilateral anterior temporal lobes (ATLs; Grönholm et al. 2005), and fusiform gyrus (Breitenstein et al. 2005) with elevated responses during training.

To fully understand native vocabulary acquisition and recovery of vocabulary in aphasia, investigation of meaningful real-world items with native language names would be ideal. A potentially suitable approach comes from a series of MEG and aphasiological studies that used the “Ancient Farming Equipment” learning paradigm, which provides a line drawing, a novel Finnish name, and a description of how the item is used (cf. Laine and Salmelin 2010). However, to fully elucidate the networks supporting word acquisition and allow charting of the neocortical transfer proposed by the CLS, a longer-term strategy is required.

In the present study, we generated a direct evaluation of the CLS with respect to native vocabulary acquisition, including the role of semantic learning. Accordingly, we used fMRI to investigate the interaction between episodic and semantic neural networks that underlie native novel vocabulary learning, and how these processes differ from long-standing fully consolidated words. Healthy, older participants were recruited for comparability with aphasic patient samples and due to increases in word-finding difficulties in normal aging (Burke and Shafto 2008). Participants were trained on novel native words for 3 weeks, before performing both picture naming (of previously known items, untrained/unknown items, and select trained items, which had been learned successfully per participant) and semantic judgment tasks in the scanner (i.e., names had to be learned sufficiently well for speech production rather than simply above-chance memory recognition). We also adopted this method and learning target as it directly mimics those found in rehabilitation of aphasic word-finding difficulties (where patients aim to re-establish meaningful, native vocabulary through multiple learning sessions, extending over several weeks). Consequently, not only does the current study provide information about native vocabulary acquisition in the healthy brain, but it may also give important clues about the neural bases of successful aphasia rehabilitation by providing a baseline for the same analysis in patients with aphasia.

We predicted that at a whole brain level, naming of newly trained, less consolidated words (for a maximum of 3 weeks, e.g., echidna, dilruba, binnacle) would rely on the episodic/MTL areas as described by the first stage of the CLS. Whereas naming of already known, highly consolidated words (e.g., dragonfly, xylophone, hairdryer) would rely on the language network, that is, the neocortical second stage of the CLS. We used behavioral measures of naming accuracy and RTs to measure how well learned and consolidated the newly learned items were. For the newly learned vocabulary, we predicted that there would be a positive correlation between ROIs in the episodic network, namely the bilateral hippocampi and left inferior parietal lobe (IPL), and longer RTs (i.e., for items that were not as well consolidated). We predicted the opposite would occur with ROIs in the language network, with more blood oxygen level-dependent (BOLD) activity in these regions correlating with quicker RTs (i.e., reflecting the gradual shift from episodic/MTL regions to neocortical ones for the most consolidated items). In contrast, for naming the established items, BOLD activity within the MTL/episodic regions would not be expected to have any significant correlations with performance measures, as this vocabulary should be well consolidated into the language system and thus rely on the language network alone. Finally, we considered the relationship between initial consolidation efficacy with longer-term retention of the newly acquired vocabulary. Specifically, we tested the hypothesis that the items which were less well consolidated after initial learning (as indexed by their higher reliance on the MTL/episodic network) would be less well retained after 6 months, whereas items that were better consolidated (as indexed by their higher activation of the language network) would be better retained.

In this study, we explored the following questions: 1) Does vocabulary acquisition follow the CLS framework of learning? 2) Does involvement of the episodic system when naming newly trained words correlate with worse performance, and does involvement of the semantic-language system when naming newly trained words correlate with better performance? 3) If so, do these correlations significantly differ from naming previously known items?

## Materials and Methods

### Participants

Twenty older, healthy native English speakers were recruited (12 females, age range 46–77 years, mean (M) age 63.90, standard deviated (SD) 8.82). All participants were right-handed, with normal or corrected-to-normal vision, no history of neurological disease, dyslexia, or contraindications to MRI scanning. The Addenbrooke’s Cognitive Examination Revised was used to screen for dementia, with a cutoff score of 88. Capacity for verbal learning was tested with the California Verbal Learning Test. All participants gave informed consent before participating, and the study was approved by a local National Health Service ethics committee.

### Stimuli

There were three sets of stimuli items (for a full list, see Supplementary Table 1). All sets contained real-world items including mammals, fish, birds, tools, food, clothing, and toys. Two sets included unfamiliar items with very low word frequency names. These items were drawn from the British National Corpus (BNC Consortium 2007), a 100 million word text corpus. One set was used for training, while the other remained as an untrained baseline set. The trained and untrained sets were counterbalanced across participants. The third set contained familiar items. These items were drawn from the International Picture Naming Project. Items were selected that could be named accurately (85–100%), with low word frequency and longer RTs (>1000 ms) to select less easily named items. All stimuli were below a word frequency of 100 words per 100 million and had high name agreement. For the baseline task, the item images for the known, trained, and untrained sets were phase scrambled. In the picture naming task, fMRI stimuli were single high quality, colored photographs with a white background. In the semantic decision task, the fMRI stimuli were presented as an orthographic written name, in black text on a white background.

### Procedure

**Figure 1:**
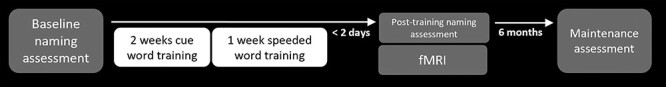
Timeline of study stages.

There were five stages: baseline naming assessment, word training, post-training behavioral assessment, functional imaging data collection, and maintenance naming assessment (Fig. 1). Participants were tested on all items before training. Stimuli sets were tailored to each participant so that all known items could be named, and all untrained and to-be-trained items could not be named prior to training. Participants undertook fMRI scanning within 2 days of finishing training. Only items which had been successfully learned, demonstrated in the post-training naming assessment, were used in the fMRI trained condition (therefore, there were different stimuli sets per participant for the trained condition). To assess maintenance, participants were tested on learned items between 5 and 6 post scanning, without interim training.

### Behavioral Training

Participants received self-guided, at-home training on new words and the related semantic information. Training took place for up to 45 min a day, 4 days a week for 3 weeks. In the first 2 weeks, participants received cue training. In the third week, participants received speeded training.

Items were presented via an interactive PowerPoint presentation. Visual Basic for Applications was used to store cue choice, time on task, and accuracy data. In weeks 1 and 2, cue training took place daily. A novel picture was shown, with the name both in orthographic and audio forms. Participants were instructed to listen to the name and repeat it out loud. After all items had been repeated, the cue training began. Participants were instructed only to use cues when they needed one and reminded they would be tested on the semantic information. The training was designed to allow healthy participants to choose the level of cue they thought they would need to be correct on each trial. This interactive and self-determined approach was chosen to make the training feel challenging, engaging and reduce boredom. 

The cue training was commonly used in standard speech and language therapy (Nickels 2002; Abel et al. 2005; Pohl et al. 2017). Participants saw a picture of an item with a choice of four cues, or the option to name the item with no cues. Participants could use as many cues as they like, in any order. There were four increasing cues. First, a picture plus a written descriptive semantic cue. Second, the picture plus the first name phoneme. Third, the first and second name phonemes were cued. The fourth cue was the whole name. All cues were given both orthographically and audibly. The semantic cue was formed in the same way for each item, initially with the geographical origins, then an identifying feature, followed by a broader semantic cue. For example, an ankus was “An Indian hooked tool used to handle and train elephants.”

After each naming attempt, the whole correct word was given. Participants were asked to indicate whether they named each item correctly or not. Participants then indicated whether the item was European or not. The initial training set was 10 items. When participants were able to name 70% of the presented items with no cue, then another 10 items were added to the set, incrementally up to 50 items.

In the third week of training, the learned items were used in a novel repeated increasingly speeded presentation (Conroy et al. 2018) learning environment. Participants were instructed that the computer would present an item for a short time, and they needed to name the picture before a specified time limit. When participants reached a success rate of 70% at a target speed, the timing was incrementally reduced from 1.8 to 1.4 s, to 1 s. When participants beat the 1 s target for 70% of items, the set size was increased by 10 items and the timing was reset to 1.8 s.

We assessed participants’ learning using a post-training assessment of trained items in the absence of cues. Only successfully named items were used during the fMRI naming task (trained vocabulary condition; *M* = 45 items), creating participant-specific trained condition naming sets. The fMRI session took place on the same day as the post-training assessment.

### Neuroimaging Acquisition

All scans were acquired on a 3T Phillips Achieva scanner, with a 32-channel head coil with a SENSE factor of 2.5. High-resolution, whole-brain, structural images were acquired including 260 slices with the following parameters: time repetition (TR) = 8.4 ms, time echo (TE) = 3.9 ms, flip angle = 8 degrees, field of view (FOV) = 240 × 191 mm, resolution matrix = 256 × 206, voxel size = 0.9 × 1.7 × 0.9 mm.

We opted to use a triple gradient echo EPI sequence in order to improve the signal-to-noise ratio, particularly in the ATLs where traditionally there are issues of EPI signal dropout and distortion (Poser et al. 2006; Halai et al. 2014, 2015). All functional scans were acquired using an upward tilt up to 45 degrees from the AC–PC line to reduce ghosting artifacts from the eyes into the temporal lobes. The sequence included 31 slices covering the whole brain with TR = 2.5 s, TE = 12, 30 and 48 ms, flip angle = 85 degrees, FOV = 240 × 240 mm, resolution matrix = 80 × 80, and voxel size = 3.75 × 3.75 × 4 mm.

All stimuli were presented electronically using E-Prime 2.0 software (Psychology Software Tools). The block order was pseudo-randomized optimized for statistical power using OptSeq (http://surfer.nmr.mgh.harvard.edu/optseq/). Verbal responses were recorded using a fiber optic microphone for fMRI (FOMRI; Optoacoustics) with noise-canceling. Participants were instructed to speak “like a ventriloquist” to reduce motion artifacts.

Participants performed two tasks during imaging acquisition, one of which is the focus of a separate study. For this study, a picture naming task comprised a block design with four conditions: known, trained, untrained, and baseline. In the known condition, participants overtly named familiar items (e.g., umbrella). In the trained condition, participants named newly learned items (e.g., echidna). If participants could not remember an item name they responded: “don’t know.” If the item was novel (untrained condition), participants also responded “don’t know.” Similarly, participants responded “don’t know” to phase-scrambled stimuli from the other conditions as the baseline condition. The task included two trial speeds but the results did not differ across these conditions; therefore, data were collapsed across this manipulation. In the standard speed condition, each 1900 ms trial consisted of a fixation cross for 700 ms, followed immediately by the target image in the middle of a white screen for 1200 ms. With five items per block, each block lasted 9.5 s. We also included eight rest blocks per run, which were jittered to have an average length of 9.5 s. With 32 task blocks and 8 rest blocks per run, the total run time was 6 min and 33 s. In the slower condition, each trial lasted 3700 ms and consisted of a fixation cross for 700 ms, followed by the target image for 3000 ms. Only three items were presented per block and each block lasted 11.1 s. As before, eight jittered rest blocks were included with an average length of 11.1 s. With 32 task blocks and 8 rest blocks, the total run time was 7 min and 4 s.

The second task, the focus of a separate study, required participants to make semantic decisions. This included three blocked conditions: trained, untrained, and baseline. In the trained and untrained conditions, participants responded “Yes” or “No” or “Don’t Know” to the semantic question “Is it European?” In the baseline task, participants responded “Up” to an ascending alphabetical sequence “ABCD” or “Down” to a descending alphabetical sequence “DCBA.” As above, we used two trial speeds but found no differences between conditions; therefore, data were combined. In the standard speed condition, a fixation cross was displayed for 700 ms, followed immediately by the target image for 1200 ms (total trial = 1900 ms). There were five trials per block each lasting 9.5 s, with six jittered rest blocks averaging to 9.5 s. The total run time was 6 min and 33 s, which included 24 task and 6 rest blocks. In the slower condition, displayed the target image for 3000 ms (total trial = 3700 ms). A total of 24 task blocks were used with three trials per block (11.1 s) and six jittered rest blocks averaging to 11.1 s (total run time = 7 min and 4 s).

### Neuroimaging Preprocessing and Analysis

T1 data was pre-processed using the FMRIB Software Library (FSL, version 6.0.0; http://fsl.fmrib.ox.ac.uk/fsl/fslwiki/, Woolrich et al. 2009). Brain tissue was extracted from the structural images (BET; Smith 2002), and an initial bias-field correction was applied using FSL’s anatomy pipeline (FAST; Zhang et al. 2001), excluding subcortical segmentation as this was performed with BET. Registration to standard space was performed in FSL with FLIRT and FNIRT (Woolrich et al. 2009; Patenaude et al. 2011) and segmentation with FAST (Zhang et al. 2001). Despiking and slice time correction were applied to the functional data in the AFNI neuroimaging suite (v19.2.10; Cox 1996; Cox and Hyde 1997; 3dDespike; 3dTshift). Combined normalization, co-registration, and motion correction parameter sets were applied to each functional echo in FSL. Functional data were optimally combined, taking a weighted summation of the three echoes, using an exponential T2^*^ weighting approach (Posse et al. 1999) and regression analysis. Functional runs were also combined and denoised using multi-echo independent component analysis (Kundu et al. 2012, 2013) using the tool meica.py (v3.2) in AFNI (Cox 1996; Cox and Hyde 1997). The denoised time series were normalized to standard space using FNIRT warps, then smoothed.

Statistical whole brain and ROI analyses were performed using SPM12 (Wellcome Trust Centre for Neuroimaging) and MarsBaR. ROIs were based upon previous literature. MTL structures, including bilateral hippocampi, are critical for episodic memory, as evidenced by hippocampal amnesia (Dickerson and Eichenbaum 2010). However, episodic memory processes also involve the IPL, despite parietal lesions not resulting in episodic memory deficits (Cabeza et al. 2008). The left inferior frontal gyrus (IFG) is considered critical in speech production and semantic processes (Blank et al. 2002; Hickok and Poeppel 2007; Lazar and Mohr 2011; Price 2012). The middle temporal gyrus (MTG) is activated during semantic processing (Binder et al. 2009; Visser et al. 2012; Noonan et al. 2013; Jackson 2021), and focal damage is associated with semantic deficits (Dronkers et al. 2004). The specific co-ordinates for these ROIs were derived by conducting a Neurosynth (Yarkoni et al. 2011) fMRI meta-analysis using two search terms: “episodic memory” (bilateral hippocampi; Montreal Neurological Institute [MNI]: −28 −14 −15, 29 −14 −15 and left IPL MNI: −47 −64 34), and “language” (left IFG MNI: −46 28 10 and left MTG MNI: −52 −42 0).

Furthermore, we included a left ventral anterior temporal lobe (vATL) ROI (MNI: −36 −15 −30) taken from a key reference (Binney et al. 2010). The vATL is often missed in fMRI studies using typical echo times of >30 ms at 3T due to signal dropout. However, there is clear evidence from the neuropsychology literature and semantic dementia patients that the vATLs are important for semantic cognition patients (Rogers et al. 2004; Patterson et al. 2007; Lambon Ralph 2014; Lambon Ralph et al. 2016). Indeed, there is growing evidence that fMRI protocols optimized for signal detection in areas of magnetic susceptibility can identify vATL areas during semantic processing (Devlin et al. 2000; Halai et al. 2014, 2015; Jackson et al. 2016; Rice et al. 2018).

RTs for ROI analyses were calculated from onset of stimulus and were *z*-scored to account for any variance due to time on task. RTs were *z*-scored by condition to enable analysis of within-condition RT variance.

## Results

### Behavioral Data

Participants spent a mean of 4.3 h training (SD = 0.8) over an average of 12 sessions. Participants successfully learned the novel vocabulary with an average gain of 81% (SD = 10.73) outside the scanner. Inside the scanner, in the trained condition, participants were presented with only items they had successfully learned during training, ascertained by a post-training behavioral picture naming task. Participants had an average of 88% (SD = 12.0) accuracy on these participant-specific trained items in scanner and an average RT of 1054 ms (SD = 203.5) in scanner. Participants also successfully learned semantic information about these successfully trained novel items, with an average gain of 83% (SD = 12.92). This level of variation in semantic knowledge of the new items (“Is it European?”) demonstrates that there was a continuum of semantic consolidation between participants in the trained items. As would be expected, naming accuracy for already-known (pre- and post-training) items during scanning was high (*M* = 98%, SD = 2.2), with a mean RT of 1020 ms (SD = 148.5). The naming latency for the newly learned and previously known items was not significantly different (*t*(19) = −.396, *p* = 0.696), indicating effectiveness of the training.

To explore the effect of semantic knowledge on word learning, correlations were performed between accuracy in the semantic judgment task (“Is it European?”), naming accuracy, and subsequent maintenance of naming accuracy. There was a significant positive correlation between naming and semantic accuracy, with age at scan added as a controlled variable (*r*(20) = 0.912, *P* = 0.000). Additionally, there was a significant correlation between learning of the semantic cues and overall maintenance of learned, trained items (*r*(20) = 0.66, *P* = 0.002).

### Whole Brain Results

**Figure 2:**
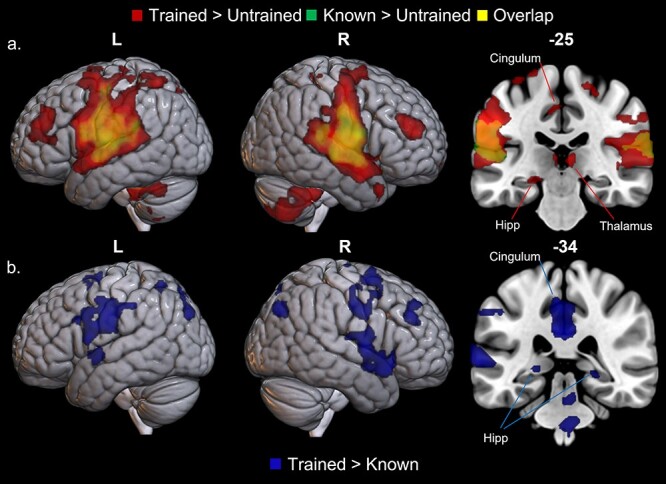
Whole brain BOLD activation of picture naming. (*a*) Trained minus untrained items (red) and known minus untrained items (green); yellow = overlap. (*b*) Trained minus known items (blue). Images thresholded at *P* < 0.001 voxel height, FWE-cluster corrected *P* < 0.05. L, left; R, right; Hipp, hippocampus; Thal, thalamus.

The results of the whole brain analyses for the picture naming task are reported in Table 1, where three contrasts were created: 1) trained > untrained, 2) known > untrained, and 3) trained > known. There were no significant clusters of activation for the opposing contrasts: untrained > trained, untrained > known, and known > trained. There was a similar pattern of activation between the contrasts, where large bilateral language areas were identified. There was, however, greater and more extensive activation for the trained condition, including the hippocampus in both the trained > untrained and trained > known contrasts (Fig. 2).

**Table 1:** Clusters significant at *P* < 0.001 voxel height and *P* < 0.05 FWE cluster correction for picture naming trained, known and untrained items

Contrast	Region of activation	Peak region	Cluster size	Peak MNI			*T*	*Z*
				*x*	*y*	*z*		
Trained > untrained	L pre/postcentral gyri, SMG,	L postcentral	42 691	−56	−14	18	11.00	6.09
	STG, IPL, hippocampus, IFG	R postcentral		60	−14	18	10.44	5.95
	(p. op), R postcentral gyrus	L precentral		−48	0	22	10.11	5.92
	R parahippocampal gyrus,	R parahipp.	979	24	10	−22	5.90	4.41
	temporal pole	R temporal pole		44	22	−32	5.68	4.29
		R parahipp.		22	14	−32	5.54	4.35
	L/R dorsal striatum, thalamus	L caudate	957	−12	−2	14	5.69	4.29
		R thalamus		4	−24	8	5.62	4.26
		L thalamus		−12	−8	14	5.32	4.08
	R MFG	R MFG	819	42	46	22	6.39	4.61
		R MFG		36	36	33	6.09	4.48
		R MFG		28	26	37	4.89	3.89
	L amygdala, orbitofrontal cortex	L amygdala	799	−16	−2	−12	5.97	4.44
		L SOG		−18	44	−16	5.71	4.30
		L MOG		−2	52	−10	5.42	4.12
Known > untrained	L pre/postcentral gyri,	L postcentral	4289	−60	−12	16	7.71	5.13
	transverse temporal gyrus,	L Heschl’s gyrus		−48	−16	8	6.59	4.70
	SMG	L STG		−60	−20	8	6.50	4.66
	R postcentral gyrus, STG,	R postcentral	3078	60	2	16	8.46	5.39
	SMG, temporal pole	R postcentral		64	−10	18	6.84	4.80
		R STG		60	−18	2	6.27	4.56
	L cerebellum	L cerebellum	426	−20	−62	−22	5.69	4.30
	R cerebellum	R cerebellum	367	12	−62	−16	5.63	4.26
Trained > known	L/R precuneus, cuneus,	L cuneus	26 037	−4	−74	26	9.02	5.56
	parahippocampal gyrus,	L calcarine		−12	−66	20	8.86	5.51
	hippocampus	L calcarine		26	−64	18	8.43	5.37
	L/R OFC, L insula, L IFG (p. tri),	L mid orbital	8968	−4	54	−6	9.86	5.80
		L insula		−34	22	2	7.96	5.22
		L IFG (p. tri)		−22	48	−16	7.52	5.06
	R insula, temporal pole, IFG	R insula	6388	42	22	−2	8.41	5.37
	(p. tri), MFG	R MFG		36	38	28	6.56	4.69
		R MFG		26	16	46	6.45	4.64
	R AG	R AG	424	44	−78	40	5.60	4.25
		R AG		38	−52	40	4.81	3.85

Note: Up to three strongest peaks listed per cluster, peak MNI = *x*, *y*, *z*. L, left; R, right; SMG, supramarginal gyrus; STG, superior temporal gyrus; IFG op, inferior frontal gyrus pars opercularis; IFG tri, inferior frontal gyrus pars triangularis; OFC, orbitofrontal cortex; AG, angular gyrus; parahipp, parahippocampal gyrus; SOG, superior orbital gyrus; MOG, middle orbital gyrus.

### ROI Analysis

**Figure 3:**
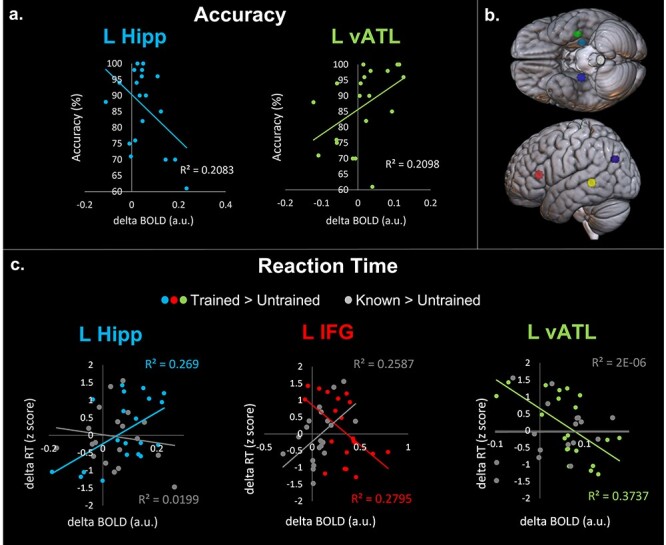
(*a*) Significant correlations of post-training percentage accuracy of trained items versus average BOLD for trained > untrained contrast. (*b*) Spherical 6 mm ROIs: right hippocampus (navy; MNI: 28 −14 −15), left hippocampus (cyan; MNI: −28 −14 −15), left IPL (purple; MNI: −47 −64 3), left IFG (red; MNI: −46 28 10), left anterior temporal lobe (green; vATL, MNI: −36 −15 −30), left MTG (yellow; MNI: −52 −42 0). (*c*) Significant correlations between contrast estimates (colored; trained > untrained, gray; known > untrained) and normalized in-scanner RT per participant per condition.

To explore a core hypothesis arising from the CLS theory (a division of labor between MTL vs. cortical regions), behavioral data were correlated with activity in a priori ROIs related to episodic memory (bilateral hippocampi and left IPL) and semantic memory (IFG, left MTG, and left ATL; Fig. 3*b*). There were no significant correlations between semantic behavioral performance and a priori ROIs.

In the initial exploratory analysis, for the trained > untrained picture naming contrast (whereby participants named pictures of newly learned items, vs. responding verbally to phase-scrambled images), we found a positive correlation between the left hippocampus and longer RTs (*r* = 0.519, *P* = 0.019; Fig. 3*c*). Conversely, we observed inverse correlations in semantic areas located in IFG (IFG; *r* = −0.528, *P* = 0.017; Fig. 3*c*) and ATL (*r* = −0.611, *P* = 0.004; Fig. 3*c*), where greater activation was related to quicker performance, suggesting they had deeper consolidation in the corresponding neocortical regions. There were no further correlations between trained > untrained BOLD and naming RTs. In the known > untrained contrast, there was a significant correlation between RT and left IFG BOLD activity (*r* = 0.509, *P* = 0.022; Fig. 3*c*). There were no further significant correlations between known > untrained BOLD activity and RT, including the left hippocampus (*r* = −0.106, *P* = 0.656) and left ATL (*r* = 0.001, *P* = 0.995; Fig. 3*c*).

The key test of the CLS hypothesis is whether the trained > untrained behavioral correlations were significantly different from the known > untrained correlations, indicating differing neural networks for naming fully consolidated known items, versus less consolidated newly trained items (for a maximum of 3 weeks). The positive correlation of hippocampal activity in the trained > untrained contrast and RT, versus the weak negative correlation of hippocampal activity in the known > untrained contrast and RT, were significantly different using Fisher’s *r*-to-*z* transformation (*z* = 1.846, *P* = 0.032, adjusted *P* = 0.032). In addition, the strong negative correlation of left ATL activity in the trained > untrained contrast and RT was significantly different to the very weak positive correlation of ATL activity in the known > untrained contrast and RT (*z* = −2.25, *P* = 0.012, adjusted *P* = 0.018). Similarly, the correlation between RT and left IFG activity was significantly different in the trained > untrained and known > untrained contrasts (which displayed a negative and positive correlation respectively; (*z* = −3.348, *P* = 0.001, adjusted *P* = 0.001), Benjamini–Hochberg adjusted *P* values for multiple comparisons (Benjamini and Hochberg 1995), *P* = 0.05.

We also correlated in-scanner accuracy with BOLD activity for the trained > untrained contrast in each ROI. In the left hippocampus, individuals with greater activity showed poorer learning (*r* = −0.456, *P* = 0.043; Fig. 3*a*). Conversely, greater activity in the left ATL related to better accuracy (*r* = 0.450, *P* = 0.046; Fig. 3*a*). Previously known items could be correctly named on three separate behavioral testing occasions, therefore, there was high (*M* = 98%) accuracy on these items in the scanner, which does not provide variation for correlation with BOLD activity and therefore negates the ability to test the key hypotheses. These two correlations were significantly different to each other however, using Fisher’s *r*-to-*z* transformation (*z* = −2.85, *P* = 0.004). All other correlations for trained > untrained accuracy, and known > untrained accuracy, with the a priori ROIs were not significant.

### Maintenance Data

**Figure 4:**
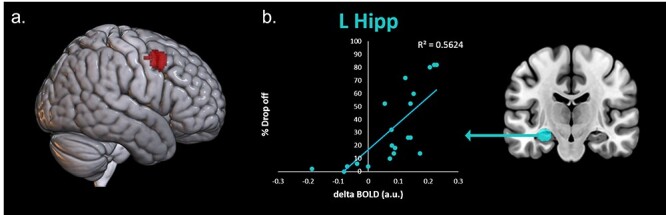
Correlations between maintenance and brain data. (*a*) Percentage trained item drop off as a covariate of interest in the trained–untrained contrast. Image thresholded at *P* < 0.001 voxel height and *P* < 0.05 FWE-cluster correction. (*b*) Significant correlation between left hippocampal activity and percentage trained item drop off.

Participants were retested on learned items 5–6 months post scanning, without interim training. Maintenance varied across participants, but overall participants named on average 73.9% (SD = 27.43) of learned words. To identify areas of BOLD activity which correlated with better or worse retention of trained items, percentage drop-off in naming performance over the maintenance period was added as a covariate of interest to the trained > untrained and known > untrained contrasts. In this covariate, higher values indicate worse retention of the trained words after the 6-month maintenance period. With percentage drop off as a covariate of interest, over trained > untrained BOLD, we identified a cluster in the right hemispheric dorsolateral prefrontal cortex (rDLPFC, peak MNI: 38 8 46, Fig. 4*a*). This indicates a correlation between more BOLD activity in the rDLPFC and greater trained item drop off (worse maintenance). There was no significant difference in the opposing direction (areas of BOLD correlating with better maintenance) or for the known untrained contrast in either direction.

To explore the predictions from the CLS framework, we obtained a correlation between maintenance and the a priori ROIs during naming of trained words (Fig. 4*b*). There was a significant positive correlation between left hippocampal activation and percentage drop off (*r*(20) = 0.605, *P* = 0.005), which suggests that individuals who were more reliant on hippocampal structures after learning were less likely to retain the newly learned vocabulary after a delay. There were no other significant correlations for trained > untrained or known > untrained contrasts.

## Discussion

Vocabulary acquisition is a lifelong process for everyday life (e.g., “coronavirus”), hobbies (e.g., “thermocline”), and careers (e.g., “temporoparietal”). Reviving vocabulary is also key for individuals with language impairment after brain injury, stroke, or dementia. This study evaluated the CLS framework (McClelland et al. 1995, 2020) for the acquisition of novel real-world vocabulary in adulthood. At one time-point post-learning, a continuum of consolidation was demonstrated, with participants responding to completely unknown and untrained words, naming successfully trained words with varying levels of semantic knowledge, and naming previously known, well-consolidated items.

The whole brain results indicate that new learning, in the trained condition, activates a combination of the typical language-semantic network, plus the hippocampal-episodic memory network. Whereas naming of well consolidated, previously known words activates the cortical language-semantic network. The ROI analyses demonstrated that activity in the left hippocampus during naming was associated with worse accuracy and slower RTs, whereas activity in the language-semantic network (left IFG, IFG, and left ATL) was associated with better accuracy and quicker RTs. Additionally, the maintenance results indicated that greater left hippocampal activity during newly trained naming was associated with greater drop off in item retention (i.e., worse maintenance).

### Complementary Learning Systems

The learning results described in this study fit within the CLS model. The CLS framework proposes a two-stage episodic-semantic account of learning: initial rapid hippocampal storage of new memories, followed typically by slower interleaved consolidation of new information alongside existing knowledge in the neocortex (McClelland 2013; McClelland et al. 2020). In this study, at the whole brain level, in both the trained > untrained and known > untrained whole brain contrasts, activated clusters formed a typical motor/language network, including the IFG. In addition, when recalling newly trained words but not when naming fully consolidated previously known words, we observed increased hippocampal activity (as observed in previous studies: Breitenstein et al. 2005, Davis et al. 2009) along with left IPL activation. Our predictions were that naming newly trained words would rely on both episodic and semantic systems, whereas naming previously known, fully consolidated words would rely on the semantic-language systems only. These whole brain analyses support this notion. ROI analyses in combination with performance allowed us to explore this hypothesis in more detail.

In the episodic ROI analyses for newly trained words, we found that left hippocampal activation was significantly associated with worse naming performance (less accuracy, longer RTs, and less maintenance of trained words after 6 months). This effect was not found for the naming of previously known items, with only a nonsignificant weakly negative correlation. These two results were in line with our predictions, specifically, that individuals reliant upon the first MTL-episodic stage of the CLS would have worse performance for the newly-acquired vocabulary. It should be noted that we only found this effect in the left hippocampus and not in the left IPL or the right hippocampus. The previous literature has demonstrated a role of the left hippocampus in vocabulary acquisition (Breitenstein et al. 2005; Davis et al. 2009). As the language network is left dominant, it is logical that the episodic system supporting language acquisition is also left dominant. The left IPL has also been indicated in previous literature during word acquisition consolidation (Pohl et al. 2017). Although there was a significant cluster of IPL activation for the trained > untrained contrast and not the known > untrained contrast, there were no significant correlations between IPL activation and behavioral performance. The functional organization of the parietal lobe is complex, and although the ROI was included as an episodic region based on previous literature (Wagner et al. 2005; Vilberg and Rugg 2008), various areas of the parietal lobe may be performing different functions, perhaps not aligning singularly with either the episodic or semantic network (Humphreys and Lambon Ralph 2015; Humphreys et al. 2020).

The neocortical areas activated by naming of newly learned items were typical of areas identified during speech production (Blank et al. 2002; Price 2012). We also identified two cortical regions associated with proficiency of naming learned items—the left vATL and left IFG. These areas are typically associated with semantic and language processing. The vATL is considered to be a trans-modal hub critical to semantic representation (Lambon Ralph et al. 2016). This proposal has strong, convergent support from multiple sources including semantic dementia patients (Warrington 1975; Jefferies and Lambon Ralph 2006; Patterson et al. 2007; Acosta-Cabronero et al. 2011), fMRI (Binney et al. 2010; Visser et al. 2012), transcranial magnetic stimulation (Pobric et al. 2007, 2010), surface cortical electrode studies (Shimotake et al. 2015), and computational modeling (Rogers et al. 2004; Chen et al. 2017; Hoffman et al. 2018; Jackson et al. 2021). Subregions of the left vATL have been associated with picture naming and speech production specifically (Sanjuán et al. 2015). The IFG has been linked to speech production, among other processes, since Broca (1861) reported a patient with loss of articulation after destruction of the IFG and surrounding cortex. Despite debate as to the exact role of subregions of the IFG in speech production (Flinker et al. 2015) and semantic control (Whitney et al. 2012; Jefferies 2013; Noonan et al. 2013; Jackson 2021), the IFG is widely recognized to be important for articulation (Blank et al. 2002; Hickok and Poeppel 2007; Lazar and Mohr 2011; Price 2012).

In these language-semantic ROIs, we found an opposite pattern of results to those found in the episodic-hippocampal analyses. When naming newly trained items, more activity in the left vATL was associated with better accuracy and shorter RTs. In contrast, there was a nonsignificant weak positive correlation between vATL activation when naming previously known words. These results align with our predictions that when individuals had better consolidated the new vocabulary (as indexed by better accuracy and shorter RTs) then this would be reflected in greater reliance upon the second neocortical stage of the CLS. This effect was also found in the left IFG, with activity during naming of newly trained items associated with quicker RTs. In addition, there was an opposite correlation of activity-behavior when naming previously known items, whereby less activity in the left IFG was associated with quicker responses. This may reflect less neural effort for production of familiar vocabulary due to well-established phonological and articulatory representations (Blank et al. 2002; Price 2010, 2012) and/or fewer semantic control requirements. The fact that we observed 1) greater neocortical activity for the trained than known words and also 2) a negative correlation between activation and performance for the trained items may well reflect the fact that not only should neocortical activation build up as the newly trained items are consolidated (and become independent of the MTL systems) but also that we know for established vocabulary from numerous language and semantic fMRI studies that there is more activation for less familiar/lower frequency words. Presumably, as proposed by many previous researchers, this reflects the fact that less frequent representations require more neural resources/longer processing times. Thus, in the “life course” of new vocabulary, one might expect an initial period in which the cortical activation builds up as the new vocabulary is cortically consolidated, but then with sufficient practice and use, the cortical representations should become more efficient and precise, thus be associated with decreasing cortical activation. This very pattern has been observed in implemented computational models of language (e.g., Chang and Lambon Ralph 2020) in which both initial vocabulary learning and relearning (after damage) are associated with an initial period of increasing unit activation and then a subsequent gradual reduction in unit activation as the underpinning (cortical) representations are more finely tuned.

It has previously been hypothesized that the CLS could apply in other domains (Davis and Gaskell 2009) and there are demonstrations in short-term pseudoword recognition (Cornelissen et al. 2004; Breitenstein et al. 2005; Mestres-Missé et al. 2007; Davis et al. 2009). Our findings complement and significantly extend these intra-learning investigations by exploring learning after full consolidation and maintenance of the new vocabulary. With 3-week training, the participants were able to name the items without cueing and make semantic decisions (i.e., more than exhibit above-chance recognition performance). Taking this body of literature together, they clearly demonstrate that the hippocampal system is critical for new learning of artificial and native vocabulary learning and that long-term consolidation reflects the gradual shift to long-term cortical representation and processing as predicted by the CLS model.

The speed of consolidation and reliance on the hippocampal-episodic network is now understood to be dependent on the strength of relationship between pre-existing knowledge and information to be learned (McClelland 2013; Kumaran et al. 2016; McClelland et al. 2020). In this study, participants learned entirely new information (items, semantics, and names). The item names are arbitrarily related to the object and their associated meaning; thus, this new knowledge is not systemically related to any pre-existing information. Therefore, the results obtained were as expected—it takes time to consolidate item names and, even after 2–3 weeks of learning, individuals remain reliant on a mixture of the hippocampal-episodic and semantic systems, rather than entirely on the cortical language-semantic system.

### Methodological Considerations

There were no significant clusters of activity for untrained > trained items. Previous literature has identified reductions of BOLD response related to word training (Nardo et al. 2017) and pseudowords versus word reading (Taylor et al. 2014). Activation can be interpreted as either engagement of the relevant systems or increased processing effort (Taylor et al. 2013). These areas of reduction can be interpreted as decreased effort associated with training. However, reductions may also signify responses to task difficulty, whereby items which are not trained are more difficult to respond to. In this study, items which were untrained were completely unknown to the participant; therefore, the task is not inherently more difficult, as participants perform the same processes of viewing an image, thinking whether they know the name, then verbally responding.

### Translational Potential

This study is also potentially informative for aphasia therapy. The neural bases of successful speech and language therapy have been rarely explored, and those studies that have done so have yielded varying results (Abel et al. 2015; Nardo et al. 2017; Woodhead et al. 2017). The methods adopted in this study were deliberately designed to mimic those used to treat word-finding difficulties, where patients aim to re-establish meaningful, native vocabulary through multiple learning sessions and vanishing phonemic cues (Abel et al. 2005), over several weeks (Dignam et al. 2016). By using the same paradigm, future studies can explore whether the neural correlates of word learning/relearning in aphasia follows the same framework. The current results would seem to imply that therapy success will depend on 1) the extent of damage to specific critical regions involved in the CLS framework and 2) damage to connectivity from the hippocampus to critical language regions. Furthermore, the majority of patients (especially those with middle cerebral artery stroke) tend to have intact hippocampus, which may be linked to the reason why patients experience initial success in learning, but long-term learning and maintenance (the goal of therapy) will relate to how well the therapy can induce relearning/stabilization of neocortical representations. If these mechanisms hold in stroke aphasia, it could have important implications for intensity and dose of speech and language therapy provision.

## Conclusion

The results of the study map the framework for word learning in the healthy older brain. In whole brain analyses, there was increased hippocampal activity when naming newly trained items, but not previously known, well-consolidated items. These results demonstrate the first stage of the CLS model, with initial hippocampal encoding. In addition, greater left hippocampal activity was associated with less accuracy, longer RTs, and less maintenance of the newly trained words. When naming well-consolidated previously known words, there was no association between hippocampal activity and performance.

The second consolidation stage of the CLS proposes a gradual shift from reliance on the MTL-episodic network towards long-term neocortical consolidation. In line with this prediction, we found that when naming newly trained words, higher levels of left IFG and vATL activation were associated with better accuracy and shorter RTs. Crucially, the associations in each ROI between BOLD activity and performance were significantly different between naming of previously known items and newly trained items. Overall, the results of this study provide evidence for both aspects of the CLS model in long-term, native word acquisition.

## Supplementary Material

Supplementary_bhab422
