# Supplementary material for: Direct neural evidence for the contrastive roles of the complementary learning systems in adult acquisition of native vocabulary
Source: Cereb Cortex. 2021 Dec 7;32(16):3392–405. doi: 10.1093/cercor/bhab422 (PMC9376875; doi:10.1093/cercor/bhab422)

Supplementary data

S1. All orthographic stimuli in the study, with 50 known, trained and untrained word sets. Trained and untrained sets were counterbalanced across participants.

| Known | Trained | Untrained |
| --- | --- | --- |
| \| Accordion \| \| --- \| \| Backpack \| \| Bagpipes \| \| Banjo \| \| Cactus \| \| Cauliflower \| \| Cheetah \| \| Cockerel \| \| Codpiece \| \| Crayon \| \| Dragonfly \| \| Dungarees \| \| Earplugs \| \| Funnel \| \| Giraffe \| \| Hairdryer \| \| Hammock \| \| Hippopotamus \| \| Kangaroo \| \| Ladle \| \| Lawnmower \| \| Mittens \| \| Peanut \| \| Pickaxe \| \| Piglet \| \| Pitchfork \| \| Pliers \| \| Protractor \| \| Pumpkin \| \| Radish \| \| Saxophone \| \| Screwdriver \| \| Seahorse \| \| Seesaw \| \| Sharpener \| \| Shoehorn \| \| Shuttlecock \| \| Snowman \| \| Spatula \| \| Stapler \| \| Starfish \| \| Tambourine \| \| Tricycle \| \| Turnip \| \| Tutu \| \| Tweezers \| \| Watermelon \| \| Wrench \| \| Xylophone \| \| Yo-yo \| | \| Aggry \| \| --- \| \| Ale-warmer \| \| Almucantar \| \| Amice \| \| Ankus \| \| Anole \| \| Armet \| \| Aspergillum \| \| Astrarium \| \| Axolotl \| \| Babirusa \| \| Bandura \| \| Bilby \| \| Binnacle \| \| Buckler \| \| Cacomistle \| \| Canezou \| \| Celesta \| \| Cervelat \| \| Chronometer \| \| Civet \| \| Colugo \| \| Corslet \| \| Cuscus \| \| Dasher \| \| Dilruba \| \| Douc \| \| Echidna \| \| Galligaskins \| \| Gelada \| \| Gerenuk \| \| Gharial \| \| Gorget \| \| Headstall \| \| Hoatzin \| \| Hyrax \| \| Kinkajou \| \| Lemming \| \| Olm \| \| Paca \| \| Pangolin \| \| Retractors \| \| Rongeur \| \| Saiga \| \| Shako \| \| Tarsier \| \| Trepan \| \| Turnshoe \| \| Ulu \| \| Vibraphone \| | \| Agouti \| \| --- \| \| Amphiuma \| \| Aquamanile \| \| Bismuth \| \| Blenny \| \| Blenny \| \| Braies \| \| Cabasset \| \| Caracal \| \| Carrasow \| \| Chapman stick \| \| Chevrotain \| \| Chevrotain \| \| Clepsydra \| \| Crinoline \| \| Dik-dik \| \| Douroucouli \| \| Dugong \| \| Electrophone \| \| Fipple \| \| Frogfish \| \| Galago \| \| Guereza \| \| Habergeon \| \| Hawkfish \| \| Hellbender \| \| Jumping shrew \| \| Kakapo \| \| Markhor \| \| Mattock \| \| Mayuri \| \| Micrometer \| \| Narwhal \| \| Nudibranch \| \| Numbat \| \| Olinguito \| \| Pelerine \| \| Pichiciego \| \| Polonaise \| \| Pooter \| \| Saolo \| \| Solenodon \| \| Stonefish \| \| Twybil \| \| Versorium \| \| Vichitra \| \| Wimple \| \| Zibellino \| |

S2. Significant clusters of activation (red) and deactivation (blue) in the known > rest picture naming contrast. P < .001 voxel level, p < .05 FWE cluster correction.


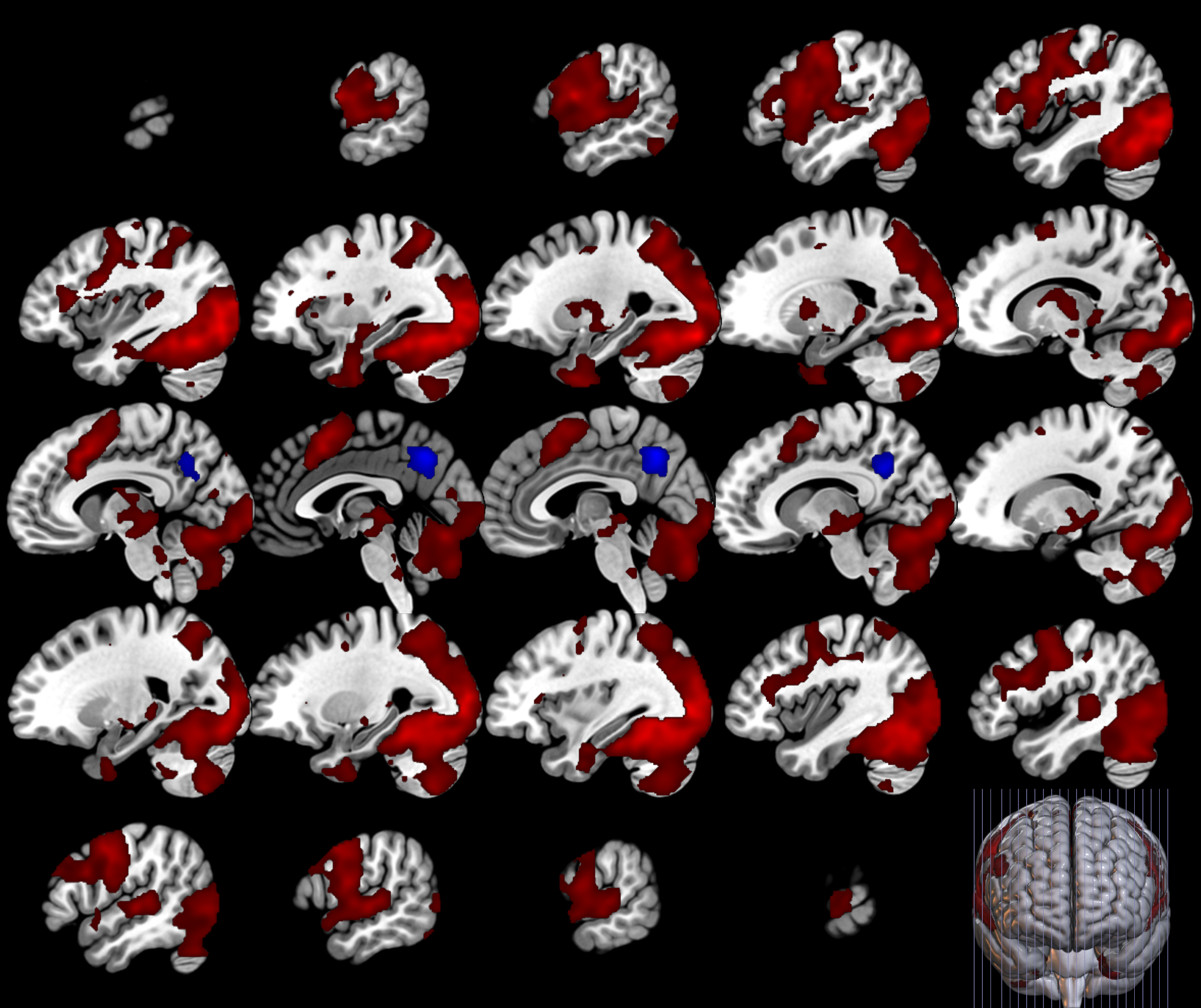


S3. Significant clusters of activation (red) in the trained > rest picture naming contrast. P < .001 voxel level, p < .05 FWE cluster correction. No significantly deactivated clusters.


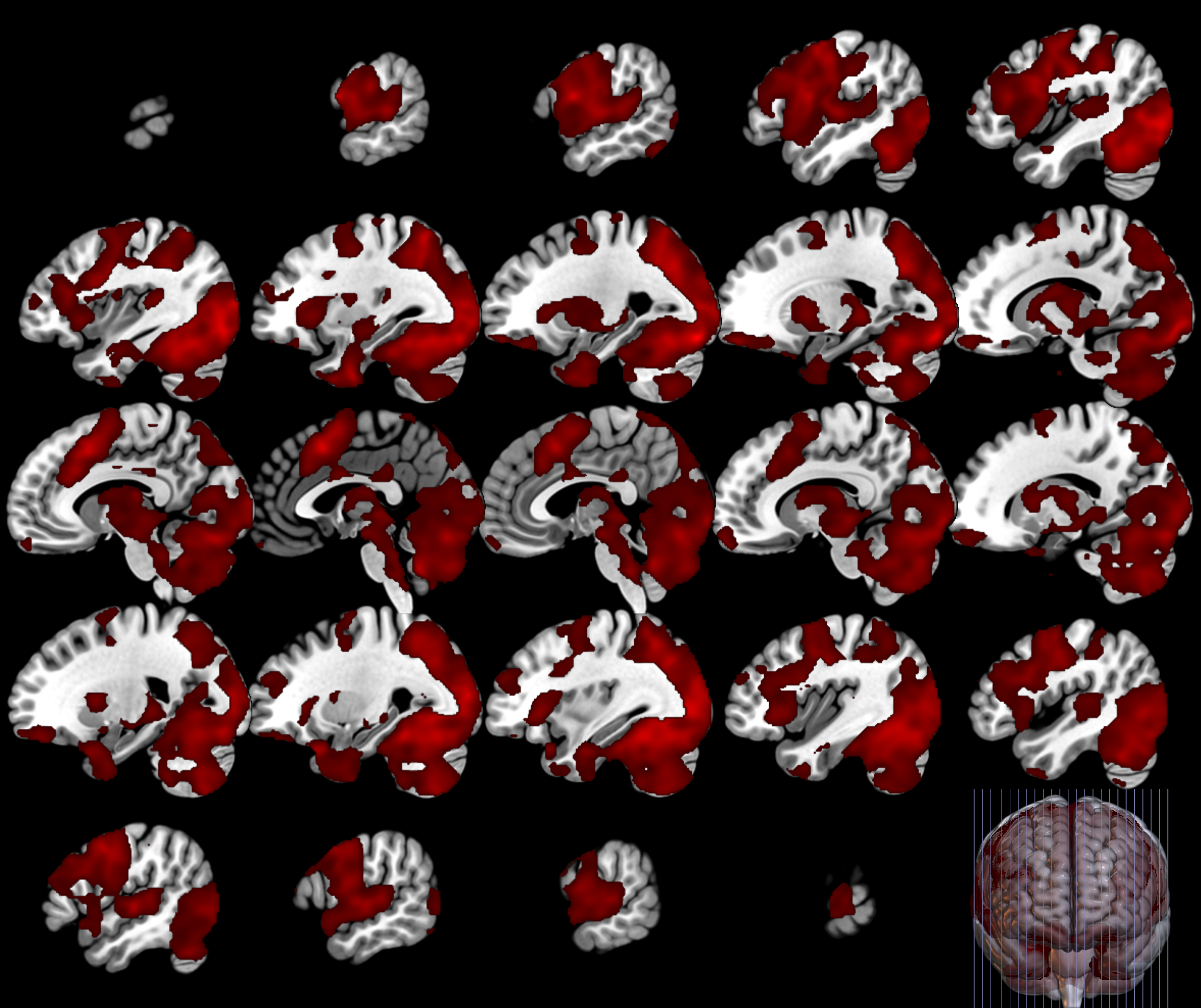

Supplement: Supplementary_bhab422 [file supplementary_bhab422.docx]
